# *BRCA1* and *BRCA2* mRNA-expression prove to be of clinical impact in ovarian cancer

**DOI:** 10.1038/s41416-018-0217-4

**Published:** 2018-08-15

**Authors:** Irina Tsibulak, Verena Wieser, Christine Degasper, Giridhar Shivalingaiah, Sören Wenzel, Susanne Sprung, Sigurd F. Lax, Christian Marth, Heidelinde Fiegl, Alain G. Zeimet

**Affiliations:** 1 Department of Obstetrics and Gynecology, Medical University of Innsbruck, Innsbruck, Austria; 2 Division of Human Genetics, Medical University of Innsbruck, Innsbruck, Austria; 3 Institute of Pathology, Medical University of Innsbruck, Innsbruck, Austria; 4 Department of Pathology, Hospital Graz Süd-West, Academic Teaching Hospital of the Medical University Graz, Graz, Austria; 5 Present Address: Division Biological Chemistry, Biocenter, Innsbruck, Medical University of Innsbruck, Innsbruck, Austria

**Keywords:** Translational research, Ovarian cancer, Molecular medicine

## Abstract

**Background:**

Mutations in *BRCA1* and *BRCA2* are associated with better survival in ovarian cancer (OC) patients due to a better response to platinum-based chemotherapy. However, the impact of the *BRCA1/2* mRNA-expression is not well characterized in OC.

**Patients and methods:**

We investigated *BRCA1/2* mRNA-expression in 12 non-neoplastic fallopian tubes and 201 epithelial OCs in relation to their clinical characteristics.

**Results:**

We found higher *BRCA1/2* mRNA-expression in OCs compared to controls (*P* = 0.011, *P* < 0.001, respectively). *BRCA1* mutated OCs exhibited lower *BRCA1* (*P* = 0.014) but higher *BRCA2* mRNA-expression (*P* = 0.001). Low *BRCA1*-expression was associated with favorable overall survival (OS) (*P* = 0.012) and low *BRCA2*-expression with better progression-free survival (PFS) and OS (*P* = 0.004, *P* = 0.001, respectively). A subgroup-analysis showed that this effect was confined only to the *BRCA1-*wildtype cancers. Cox-regression confirmed the prognostic significance of *BRCA1*-expression for OS (*P* = 0.028). Independency of the prognostic value of *BRCA2*-expression for PFS (*P* = 0.045) and OS (*P* = 0.015) was restricted to high-grade serous OCs. Fully platinum-sensitivity was characterized by lower *BRCA1/2* mRNA-expression in *BRCA1*-wildtype cancers in comparison to platinum-refractory OC.

**Conclusion:**

Our findings may reflect higher platinum-sensitivity due to reduced capacity of DNA damage repair in tissues with low *BRCA1/2*-expression. In this context, especially in *BRCA*-wildtype cancers both parameters could also be potential predictors for PARP-sensitivity.

## Introduction

*BRCA1* and *BRCA2* are tumor suppressor genes that are involved in cell growth inhibition, apoptosis, regulation of gene transcription and DNA damage repair through homologous recombination.^1^ Thus, germ line mutations in *BRCA* genes are considered to be associated with cancer susceptibility, especially with an increased risk of developing ovarian and breast cancer.^2^

Epithelial ovarian cancer (OC) is one of the leading causes of cancer death in women in the western world.^3–5^ Approximately 5–10% of all epithelial OC are hereditary and at least two-third of them are due to *BRCA1*/*2* mutations.^6,7^

The current opinion is that OC patients who carry a pathogenic *BRCA* mutation show better survival rates possibly due to a better response to platinum-based chemotherapy and inhibitors of poly(ADP)-ribose polymerase (PARP).^8,9^ However, there are also conflicting data, showing worse survival in hereditary OC cases or no significant difference in survival rates between patients with *BRCA* associated and sporadic epithelial OC.^10–12^ Such discrepancies could be due to different duration of follow-up, different histological type or a death caused by secondary malignancies.

Besides *BRCA* mutation-status, the expression of these genes could contribute to the tumor pathogenesis and therapeutical response. Currently there is lack of data on *BRCA1*/*2* expression and their clinical significance in epithelial OC patients. The implication of *BRCA*-expression in *BRCA*-wildtype epithelial OC is also poorly studied but could be clinically relevant. We wondered whether the expression of *BRCA1*/*2* on the transcriptome level could be a reliable predictor for platinum response and thus for the clinical outcome in OC patients.

In our study, we evaluated *BRCA1/2*-mRNA-expression in frozen tissues of 201 epithelial OC patients. We analyzed progression-free survival (PFS), overall survival (OS), the association between the *BRCA*-expression and mutation-status and methylation-status as well as FIGO stage and achievement of a complete resection during debulking surgery.

## Patients and methods

### Patients and samples

Ovarian tissue samples from 201 patients with OC obtained at primary debulking (patients were 24–90 years old; median age at diagnosis was 62 years) and non-neoplastic tubal tissues from 12 patients obtained by elective salpingo-oophorectomy for benign conditions (patients were 30–73 years old, median age: 50 years) were collected and processed at the Department of Obstetrics and Gynecology of the Medical University of Innsbruck between 1989 and 2015 as described recently.^13^ Systemic treatment of OC patients consisted of six adjuvant cycles of platinum-based chemotherapy. We used a categorization which defines “platinum-refractory” as disease progressing during therapy or within one month after the last dose, “platinum-resistant” as disease progressing within 6 months, “partially platinum-sensitive” as disease progressing between 6 and 12 months, and “platinum-sensitive” as disease progressing with an interval of more than 12 months. Written informed consent was obtained from all patients before enrollment. The study was reviewed and approved by the Ethics committee of the Medical University of Innsbruck (reference number: AN2015-0038 346/4.17) and conducted in accordance with the Declaration of Helsinki. The median observation period of all patients was 1.6 years (0.03–26.4 years) regarding the progression-free survival and 3.6 years (0.1–26.4 years) concerning the median overall survival. Clinicopathological characteristics are shown in Table 1.

**Table 1:** Association of *BRCA1 and BRCA2* mRNA-expression and mutations with clinicopathological characteristics in 201 ovarian cancer patients

Variable	Number (%)	mRNA expression values (arbitrary units)				Somatic mutations						DNA methylation status		
		*BRCA1*		*BRCA2*		*BRCA1*			*BRCA2*			*BRCA1*		
		Median (IQR)	*P* value	Median (IQR)	*P* value	Non-mutated (%)	Mutated (%)	*P* value	Non-mutated (%)	Mutated (%)	*P* value	Unmethylated (%)	Methylated (%)	*P* value
*Age*														
≤62.3 years	101 (50%)	0.81 (0.53–1.30)	0.491	3.72 (2.14–6.15)	0.875	73 (73%)	27 (27%)	**0.001**	95 (94%)	5 (5%)	0.743	88 (88%)	12 (12%)	0.504
>62.3 years	100 (50%)	0.86 (0.55–1.37)		3.65 (2.47–6.01)		90 (90%)	9 (9%)		93 (93%)	6 (6%)		90 (91%)	9 (9%)	
*FIGO stage*														
I/II	50 (25%)	0.91 (0.57–1.35)	0.361	3.22 (2.08–4.87)	0.089	42 (84%)	7 (14%)	0.426	48 (96%)	1 (2%)	0.219	48 (96%)	2 (4%)	0.081
III/IV	151 (75%)	0.80 (0.53–1.30)		4.02 (2.67–6.17)		121 (80%)	29 (19%)		140 (93%)	10 (7%)		130 (87%)	19 (13%)	
*Tumor grade*														
1	14 (7%)	0.52 (0.43–1.01)	0.099	2.07 (1.19–2.92)	**<0.001**	12 (86%)	2 (14%)	0.789	14 (100%)	0 (0%)	0.624	13 (93%)	1 (7%)	**0.004**
2	90 (45%)	0.85 (0.56–1.26)		3.55 (2.22–5.35)		71 (80%)	18 (20%)		84 (94%)	5 (6%)		87 (97%)	3 (3%)	
3	95 (47%)	0.82 (0.58–1.55)		4.51 (3.00–7.30)		78 (83%)	16 (17%)		88 (94%)	6 (6%)		76 (82%)	17 (18%)	
n.a.	2 (1%)	–				–	–					–	–	
*Residual disease*														
Macroscopically tumor-free	100 (50%)	0.87 (0.58–1.34)	0.261	3.36 (2.04–5.16)	**0.032**	82 (82%)	17 (17%)	0.588	92 (92%)	7 (7%)	0.399	94 (95%)	5 (5%)	**0.013**
Any tumor residual	95 (47%)	0.78 (0.51–1.33)		3.99 (2.82–6.23)		75 (79%)	19 (20%)		90 (95%)	4 (4%)		79 (84%)	15 (16%)	
n.a.	6 (3%)													
*Histology*														
HGSOC	129 (64%)	0.79 (0.55–1.31)	**0.025**	4.13 (2.59–6.16)	*0.005*	102 (79%)	25 (19%)	0.848	117 (91%)	10 (8%)	0.314	114 (89%)	14 (11%)	0.993
LGSOC	12 (6%)	0.48 (0.38–0.67)		2.05 (1.16–2.87)		10 (83%)	2 (17%)		12 (100%)	0 (0%)		11 (92%)	1 (8%)	
Endometrioid	45 (22%)	1.05 (0.68–1.48)		4.44 (2.91–6.40)		37 (82%)	8 (18%)		44 (98%)	1 (2%)		40 (89%)	5 (11%)	
Clear cell	11 (5%)	0.97 (0.74– 1.44)		2.88 (2.00–4.56)		10 (91%)	1 (9%)		11 (100%)	0 (0%)		9 (90%)	1 (10%)	
Unknown	4 (2%)	–		–		–	–		–	–		–	–	
*Ovarian cancer type*														
Type I	14 (7%)	0.52 (0.43–1.00)	**0.034**	2.07 (1.19–2.92)	**0.001**	12 (86%)	2 (14%)	0.676	14 (100%)	0 (0%)	0.342	13 (93%)	1 (7%)	0.650
Type II	183 (91%)	0.85 (0.57–1.36)		4.09 (2.63–6.16)		147 (80%)	34 (19%)		170 (93%)	11 (6%)		161 (89%)	20 (11%)	
Unknown	4 (2%)	–		–		–	–		–	–				
*BRCA1 mutation*														
Wild type	163 (81%)	0.90 (0.57–1.40)	**0.014**	3.48 (2.23–5.48)	**0.001**	–	–	–	–	–	–	141 (87%)	21 (13%)	**0.024**
Mutate	36 (18%)	0.66 (0.42–0.89)		5.92 (3.27–8.38)		–	–		–	–		35 (100%)	0 (0%)	
n.a.	2 (1%)	–		–										
*BRCA2 mutation*														
Wild type	188 (94%)	0.82 (0.53–1.29)	0.073	3.84 (2.46–6.16)	0.346	–	–	–	–	–	–	166 (89%)	20 (11%)	0.862
Mutate	11 (6%)	1.59 (0.59–2.39)		3.40 (1.97–4.18)		–	–		–	–		10 (91%)	1 (9%)	
n.a.	2 (1%)	–		–										
*BRCA1/2 mutation*														
Wild type	152 (76%)	0.88 (0.55–1.33)	0.178	3.48 (2.26–5.51)	**0.015**	–	–	–	–	–	–	131 (87%)	20 (13%)	**0.033**
Mutate	47 (24%)	0.68 (0.44–1.35)		5.14 (2.80–7.55)								45 (98%)	1 (2%)	
n.a.	2 (1%)	–		–		–	–		–	–				
*BRCA1 DNA methylation*														
Unmethylated	178 (89%)	0.88 (0.58–1.35)	**<0.001**	3.41 (2.17–5.78)	**0.001**	–	–	–	–	–	–	–	–	–
Methylated	21 (11%)	0.20 (0.14–0.46)		5.52 (3.99–8.21)		–	–		–	–		–	–	
*BRCA2 DNA methylation*														
Unmethylated	168 (100%)	0.78 (0.53–1.28)	–	3.68 (2.36–6.01)	–	–	–	–				–	–	–
Methylated	0 (0%)	–		–	–	–	–					–	–	

Bold values indicates $P \ \noexpand\lt$ 0.05

### RNA isolation and reverse transcription

Total cellular RNA extraction from and reverse transcription were performed as previously described.^13^

### Quantitative real time PCR

Primers and probes for the TATA box-binding protein (TBP; endogenous RNA-control) were used as previously described.^13^ Primers and probes for *BRCA2* [GenBank: NM_000059.3] were determined with the assistance of the computer program Primer Express (Life Technologies, Carlsbad, CA, USA). *BRCA2* forward primer: 5′-GAA AAT CAA GAA AAA TCC TTA AAG GCT-3′; *BRCA2* reverse-primer: 5′-GTA ATC GGC TCT AAA GAA ACA TGA TG-3′; *BRCA2* TaqMan probe: 5′-FAM-AGC ACT CCA GAT GGC ACA ATA AAA GAT CGA AG-3′-TAMRA. Primers and probe for *BRCA1* were purchased from Applied Biosystems (Foster City, CA, USA, Applied Biosystems Assay ID: Hs01556193_m1). PCR reactions were performed as previously described.^13^ Each experiment included a standard curve with five cDNA concentrations, a positive control sample (OVCAR-3 carcinoma cell-line), 40 patient samples and a no template control. The standard curves were generated using serially diluted solutions of standard cDNA derived from the HTB-77 carcinoma cell line. The target mRNA quantity in each sample was determined from the relative standard curve, data normalization was carried out against TBP, the endogenous RNA-control and expressed in arbitrary units corresponding to the dilution factors of the standard RNA preparation. Real-time PCR assays were conducted in duplicates for each sample, and the mean value was used for calculation.

### Mutation analysis

Genomic DNA from pulverized, quick-frozen OC specimens was isolated using the DNeasy tissue-kit (Qiagen, Hilden, Germany). Targeted NGS was performed using the TruSight Cancer sequencing panel (Illumina, San Diego, USA). The analyses were performed on the Illumina MiSequ® and the NextSeq system (Illumina, CA, USA). Mutation analysis was performed using NextGene and Geneticist Assistant softwares.

### DNA-methylation analysis

Bisulfite modification and MethyLight analysis were performed as described previously.^14^ For *BRCA1* DNA-methylation two different MethyLight PCR primer sets were used, Primers and probes for *BRCA1* were determined with the assistance of the computer program Primer Express version 2.0.0 (Applied Biosystems, Foster City, CA, USA) to produce a 86-base-pair PCR amplicon (located at +57 to +142 relative to transcription start site of *BRCA1*). Genomic DNA not treated with bisulfite (unmodified) was not amplified with the primers (data not shown). Primer sequences were: *BRCA1* forward 5′-ATC CCC CGT CCA AAA AAT CT-3′, *BRCA1* reverse 5′-TGG TAA CGG AAA AGC GCG-3′, *BRCA1* Taq Man probe 5′FAM- CAC GCC GCG CAA TCG CAA -3′-BHQ1. For *BRCA1* DNA-methylation analysis an additional MethyLight reaction was selected from literature, also primers and probes for *BRCA2* were selected from literature.^15^ Cases were scored as positive if a percentage of methylated reference (PMR) value of ≥4.0% was obtained, according to studies published in the literature.^16,17^

### Statistical analysis

To compare clinicopathological characteristics and *BRCA1/2* mRNA-expression or *BRCA1/2* mutation-status, the non-parametric Mann–Whitney *U* test or Kruskal–Wallis test or Chi-squared test were applied. The correlations between *BRCA1/2* mRNA-expression were assessed by Spearman-rank correlation analyses. Progression-free survival (PFS) was defined as the time from diagnosis of the primary to tumor to the histopathological confirmation of recurrence or metastases and overall survival (OS) as the time from diagnosis of the primary to tumor to death from any cause or to the last clinical inspection. Univariate Kaplan-Meier analyses and multivariable Cox survival analyses were used to explore the association of *BRCA1/2* expression or with PFS and OS. For survival analyses, patients were dichotomized into low and high mRNA-expression level groups by the optimal cut-off expression value calculated by the Youden’s index based on a receiver operating characteristic curve analysis for overall survival.^18^ *P*-values less than 0.05 were considered as statistically significant. Statistical analysis was performed using SPSS statistical software (version 20.0.0; SPSS Inc., Chicago, IL, USA).

## Results

**Fig. 1:**
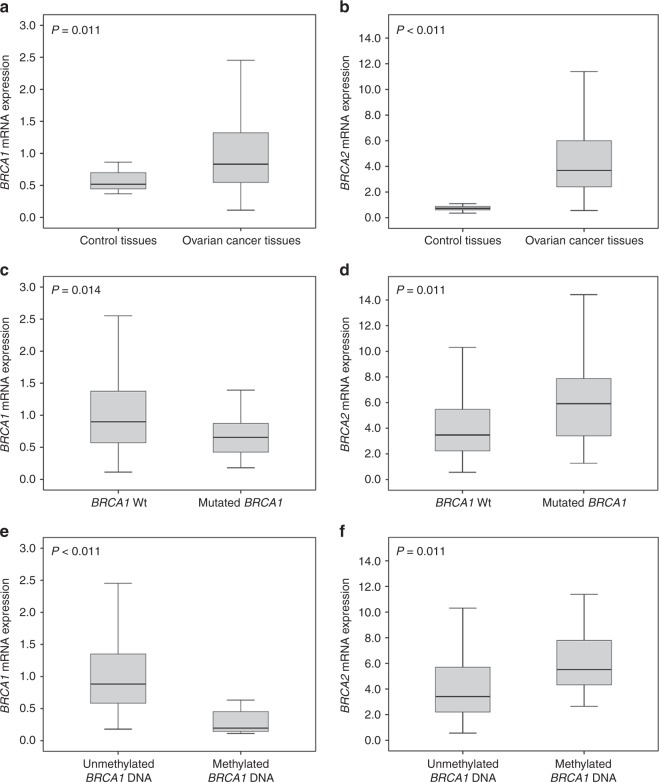
*BRCA1* and *BRCA2* mRNA-expression in ovarian tissues. **a** *BRCA1* mRNA-expression in 12 non-neoplastic fallopian tubes and 192 OC tissues, **b** *BRCA2* mRNA-expression in 11 non-neoplastic fallopian tubes and 168 OC tissues. **c** *BRCA1* and **d** *BRCA2* mRNA-expression according the *BRCA1* mutation-status. **e** *BRCA1* and **f** *BRCA2* mRNA-expression according the *BRCA1* DNA-methylation-status

We analyzed *BRCA1/2* mRNA-expression levels in 201 OC tissues and 12 non-neoplastic fallopian tubes. We found 1.6-fold higher *BRCA1* and 5.0-fold higher *BRCA2* mRNA-expression levels in OC samples in comparison to control tissues (*P* = 0.011, *P* < 0.001, Fig. 1a, b).

### Molecular and clinicopathological characteristics

Associations of *BRCA1/2* mRNA-expression with clinicopathological characteristics are shown in Table 1.

We found that *BRCA2* mRNA-expression was associated with poor tumor differentiation as it increases with tumor grade (*P* < 0.001; Table 1). Higher *BRCA2* mRNA-expression was observed in patients with any residual disease (*P* = 0.032; Table 1) in comparison to patients with no residual disease. The highest *BRCA1/2* mRNA-expression levels were identified in endometrioid OCs in comparison to the other histologic subtypes (*P* = 0.025, and *P* = 0.005, respectively; Table 1). Ninety-one percent of the patients included in this study had type II tumors (*N* = 183) which showed higher, intratumoral *BRCA1/2* mRNA-expression compared to type I tumors (*P* = 0.034 and *P* = 0.001, respectively; Table 1).

OC tissues with *BRCA1*-mutations showed lower *BRCA1* mRNA-expression (*P* = 0.014) but higher *BRCA2* mRNA-expression (*P* = 0.001) (Table 1; Fig. 1c, d) in comparison to tissues without *BRCA1* mutations. No association between *BRCA2* mutation-status and *BRCA1/2* mRNA-expression was identified (Table 1). Among 201 OC patients 36 patients (18%) presented *BRCA1*-mutations, 11 patients (6%) *BRCA2-*mutations. Interestingly, there was no correlation between *BRCA* mutation-status and any clinicopathological characteristics. In the herein investigated cohort, no differences in the expression of *BRCA-1/2*-mRNA could be revealed for the various subtypes of mutations detected in the *BRCA1/2* genes (data not shown).

As expected, we found an inverse association between *BRCA1* DNA-methylation-status and *BRCA1* mRNA-expression (*P* < 0.001; Table 1; Fig. 1e). Interestingly we observed a direct association between *BRCA1* DNA-methylation and *BRCA2* mRNA-expression (*P* = 0.001; Table 1; Fig. 1f). Eighteen percent of undifferentiated tumors (tumor grade 3, *N* = 17) and 16% of tumors from patients with any residual disease (*N* = 15) were positive for *BRCA1* DNA-methylation (*P* = 0.004, and *P* = 0.013; respectively; Table 1). Epigenetic silencing of *BRCA1* was mutually exclusive with *BRCA1* mutations (Table 1). No *BRCA2* DNA-methylation was detected in the analyzed OC tissue samples.

### Survival analysis of *BRCA1* and *BRCA2* mRNA-expression and DNA-methylation-status

**Fig. 2:**
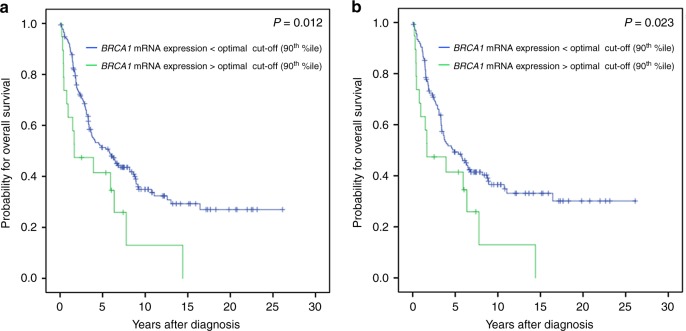
Kaplan Meier survival analysis and *BRCA1* mRNA-expression in OC patients according the 90th percentile as cut-off value. Overall survival in **a** 192 OC patients, **b** 155 OC patients with *BRCA1*-wildtype tumors

**Fig. 3:**
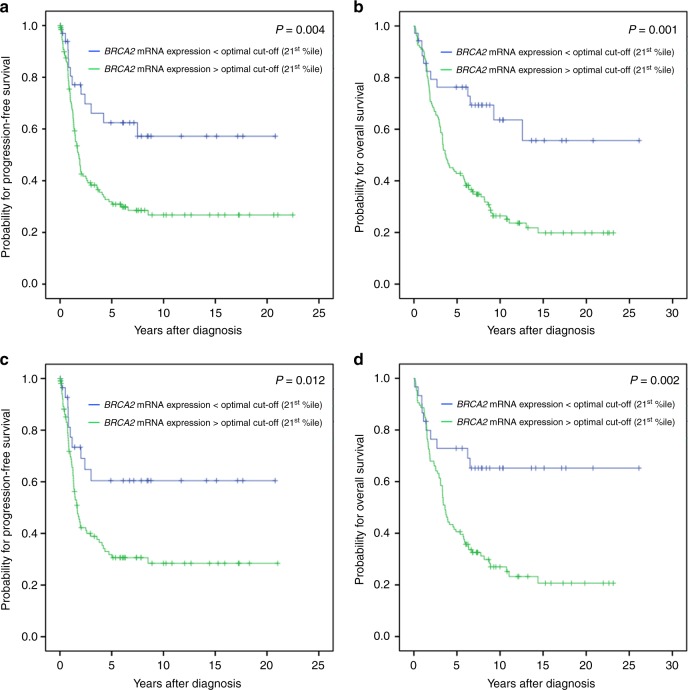
Kaplan Meier survival analysis and *BRCA2* mRNA-expression in OC patients according the 21st percentile as cut-off value. **a** Progression-free survival and **b** overall survival in 168 OC patients. **c** Progression-free survival and **d** overall survival in 136 patients with *BRCA1*-wildtype tumors

In order to investigate the prognostic value of *BRCA1/2* mRNA-expression levels we identified the optimal threshold for “high” and “low” expression using Youden’s index.^16^ Univariate survival analysis in the entire cohort showed that a lower *BRCA1* mRNA-expression (<90th percentile) was associated with a favorable OS (*P* = 0.012; Table 2A; Fig. 2a). This was also observed in the subgroups of high grade OC (*P* = 0.004; Table 2B) and high grade serous OC (*P* = 0.027; Table 2C). A detailed analysis revealed that these prognostic effects were only observed in patients with *BRCA1* non-mutated (wildtype) tumors in all patients (*P* = 0.023; Table 2A; Fig. 2b) and in high grade OC patients (*P* = 0.011; Table 2B). Lower *BRCA2*-expression levels (<21st percentile) were associated with favorable PFS and OS in the entire cohort (*P* = 0.004; *P* = 0.001; Table 2A; Fig. 3a, b), in high grade OC (*P* = 0.006; *P* = 0.002; Table 2B) and in high grade serous OC (*P* = 0.006; *P* = 0.001; Table 2C). A detailed analysis showed again the prognostic relevance of low *BRCA2* mRNA-expression only in patients with *BRCA1* non-mutated tumors. This was true for the entire patient cohort (PFS: *P* = 0.012; OS: *P* = 0.002; Table 2A; Fig. 3c, d), high grade OC (PFS: *P* = 0.022; OS: *P* = 0.005; Table 2B) and high grade serous OC (PFS: *P* = 0.016; OS: *P* = 0.001; Table 2C). No impact of *BRCA1* gene promoter methylation-status on progression-free survival and overall-survival rates was found (Table 2).

**Table 2:** Univariate survival analysis in 201 ovarian cancer patients

Variable		Progression-free survival		Overall survival	
		Median, years (95% CI)	*P* value	Median, years (95% CI)	*P* value
A					
Age	≤62.3 years	2.05 (1.47–2.63)	0.805	8.20 (5.63–10.78)	**0.006**
	>62.3 years	1.81 (1.13–2.50)		3.35 (2.68–4.02)	
FIGO stage	I/II	n.r.	**<0.001**	n.r.	**<0.001**
	III/IV	1.48 (1.10–1.86)		3.62 (3.06–4.18)	
Tumor grade	1/2	2.05 (1.23–2.87)	0.221	6.24 (2.82–9.67)	0.057
	3	1.97 (1.16–2.78)		3.62 (3.03–4.21)	
Residual disease	Macroscopically tumor-free	n.r.	**<0.001**	13.03 (n.r.)	**<0.001**
	Any tumor residual	1.25 (1.06–1.44)		2.68 (1.83–3.53)	
Histology	HGSOC	1.77 (1.35–2.18)	**0.027**	3.62 (3.13–4.12)	**0.006**
	LGSOC	n.r.		n.r.	
	Endometrioid	5.98 (n.r.)		11.06 (n.r.)	
	Clear cell	1.81 (1.10-2.53)		2.72 (n.r.)	
Ovarian cancer type	Type I	n.r.	0.068	n.r.	**0.022**
	Type II	1.91 (1.52–2.29)		3.82 (2.11–5.52)	
*BRCA1 DNA* methylation	No	2.00 (1.41–2.59)	0.850	4.54 (2.55–6.53)	0.521
	Yes	1.95 (0.46–3.45)		4.89 (2.25–7.53)	
*BRCA1* mRNA expression	Low	2.02 (1.38–2.65)	0.183	5.74 (3.63–7.85)	**0.012**
	High	0.87 (0.00–1.82)		1.66 (0.00–5.00)	
*Subgroup analysis*					
*BRCA1* non-mutated	Low	2.06 (1.03–3.10)	0.169	4.89 (2.88–6.90)	**0.023**
	High	0.87 (0.00–1.82)		1.67 (0.00–5.00)	
*BRCA1* mutated	Low	2.00 (1.60–2.39)	–	8.20 (4.52–11.88)	–
	High	–		–	
*BRCA2* mRNA expression	Low	n.r.	**0.004**	n.r.	**0.001**
	High	1.81 (1.41–2.22)		3.70 (2.78–4.62)	
*Subgroup analysis*					
*BRCA1* non-mutated	Low	n.r.	**0.012**	n.r.	**0.002**
	High	1.65 (1.19–2.11)		3.62 (3.09–4.14)	
*BRCA1* mutated	Low	7.49 (n.r.)	0.372	9.27 (n.r.)	0.463
	High	1.98 (1.62–2.34)		6.03 (0.28–11.78)	
B					
Age	≤62.3 years	1.98 (1.34–2.62)	0.590	6.86 (4.23–9.50)	**0.012**
	>62.3 years	1.77 (1.27–2.26)		3.32 (2.63–4.01)	
FIGO stage	I/II	n.r.	**<0.001**	n.r.	**<0.001**
	III/IV	1.47 (1.10–1.84)		3.43 (3.01–3.85)	
Tumor grade	2	1.90 (1.40–2.41)	0.398	5.74 (2.79–8.68)	0.177
	3	1.95 (1.20–2.71)		3.55 (3.01–4.08)	
Residual disease	Macroscopically tumor-free	5.98 (n.r.)	**<0.001**	13.03 (n.r.)	**<0.001**
	Any tumor residual	1.25 (1.06–1.44)		2.55 (1.58–3.51)	
Histology	HGSOC	1.77 (1.35–2.18)	0.056	3.62 (3.13–4.12)	**0.029**
	HGEOC	5.11 (n.r.)		8.94 (5.85–12.02)	
	HGCCOC	1.81 (1.10–2.53)		2.72 (n.r.)	
*BRCA1 DNA* methylation	No	1.91 (1.35–2.47)	0.733	3.92 (2.09–5.76)	0.531
	Yes	1.95 (0.51–3.40)		3.71 (1.65–5.77)	
*BRCA1* mRNA expression	Low	1.98 (1.34–2.62)	0.093	4.89 (2.74–7.04)	**0.004**
	High	0.87 (0.02–1.72)		1.65 (0.68–2.63)	
*Subgroup analysis*					
* BRCA1* non-mutated	Low	1.95 (1.07–2.84)	0.094	3.94 (1.94–5.94)	**0.011**
	High	0.87 (0.02–1.72)		1.65 (0.68–2.63)	
* BRCA1* mutated	Low	2.00 (1.80–2.19)	–	8.20 (3.28–13.12)	–
	High	–		–	
*BRCA2* mRNA expression	Low	n.r.	**0.006**	n.r.	**0.002**
	High	1.81 (1.40–2.23)		3.62 (2.97–4.27)	
*Subgroup analysis*					
* BRCA1* non-mutated	Low	n.r.	**0.022**	n.r.	**0.005**
	High	1.65 (1.19–2.11)		3.43 (2.96–3.90)	
* BRCA1* mutated	Low	7.49 (n.r.)	0.326	12.58 (n.r.)	0.461
	High	1.98 (1.62–2.34)		6.03 (0.28–11.78)	
C					
Age	≤62.3 years	1.81 (1.20–2.42)	0.650	5.74 (2.89–8.58)	**0.035**
	>62.3 years	1.68 (1.07–2.29)		3.32 (2.74–3.89)	
FIGO stage	I/II	n.r.	**<0.001**	7.78 (1.38–14.18)	**0.049**
	III/IV	1.47 (1.09–1.84)		3.55 (3.15–3.94)	
Tumor grade	1/2	1.65 (1.10–2.20)	0.519	3.82 (1.35–6.29)	0.257
	3	1.95 (1.49–2.42)		3.55 (3.10–3.99)	
Residual disease	Macroscopically tumor-free	3.57 (0.00–7.23)	**<0.001**	8.17 (2.26–14.08)	**<0.001**
	Any tumor residual	1.26 (1.09–1.42)		2.94 (1.91–3.96)	
*BRCA1* DNA methylation	No	1.77 (1.33–2.21)	0.943	3.62 (3.07–4.17)	0.750
	Yes	1.95 (0.92–2.99)		3.71 (2.02–5.40)	
*BRCA1* mRNA expression	Low	1.84 (1.51–2.17)	0.101	3.71 (2.73–4.69)	**0.027**
	High	0.87 (0.14–1.60)		1.65 (0.42–2.88)	
*Subgroup analysis*					
* BRCA1* non-mutated	Low	1.68 (1.20–2.17)	0.117	3.62 (3.07–4.18)	0.051
	High	0.87 (0.14–1.60)		1.65 (0.42–2.88)	
* BRCA1* mutated	Low	1.98 (1.70–2.26))	–	6.03 (1.35–10.71)	–
	High	–		–	
*BRCA2* mRNA expression	Low	n.r.	**0.006**	n.r.	**0.001**
	High	1.67 (1.31–2.03)		3.39 (3.01–3.77)	
*Subgroup analysis*					
* BRCA1* non-mutated	Low	n.r.	**0.016**	n.r.	**0.001**
	High	1.46 (1.06–1.87)		3.39 (2.87–3.91)	
* BRCA1* mutated	Low	7.49 (n.r.)	0.531	12.58 (n.r.)	0.571
	High	1.98 (1.69–2.27)		4.08 (0.00–9.78)	

The significance level (*P*) was determined by log-rank test

*HGCCOC* high grade clear cell ovarian cancer, *HGEOC* high grade endometrioid ovarian cancer, *HGSOC* high grade serous ovarian cancer, *LGSOC* low grade serous ovarian cancer, *n.r.* not reached

A: Progression free and overall survival in 201 ovarian cancer patients

B: Subgroup analysis: progression-free and overall survival in 183 high grade OC patients

C: Subgroup analysis: progression-free and overall survival in 129 high grade serous OC patients. The optimal cutoff points for *BRCA1/2* mRNA expression were calculated by the Youden’s index for overall survival (*BRCA1* expression: low/ high:</>90th %ile; *BRCA2* expression: low/ high:</>21st %ile). Bold values indicates $P \ \noexpand\lt$ 0.05

Cox-regression survival analysis confirmed the prognostic significance of *BRCA1* mRNA-expression for OS in the whole cohort (HR_death_ 2.0 (1.1–3.7), *P* = 0.028; Table 3A) but not in high grade serous OC (Table 3B). However, independency of the prognostic value of *BRCA2* mRNA-expression was approved in patients with high grade serous OC, representing 64% of the entire cohort, as well for PFS (HR_progression_ 2.4 (1.0–5.7), *P* = 0.045) as for OS (HR_death_ 2.9 (1.2–6.8), *P* = 0.015); (Table 3B).

**Table 3:** Multivariable analysis in ovarian cancer patients

Variable		Progression-free survival		Overall survival	
		HR of progression (95% CI)	*P* value	HR of death (95% CI)	*P* value
A					
Age	Low vs. high (<or>median age)	–	–	1.9 (1.3–2.9)	**0.001**
FIGO stage	I/II vs. III/IV	2.6 (1.2–5.5)	**0.013**	1.1 (0.6–2.1)	0.692
Residual disease after surgery	No vs. yes	2.7 (1.6–4.6)	**<0.001**	3.5 (2.1–6.0)	**<0.001**
Histology	HGSOC vs. Others	0.9 (0.6–1.5)	0.675	0.6 (0.4–1.1)	0.087
Ovarian cancer type	Type I vs. Type II	–	–	0.9 (0.3–2.7)	0.829
*BRCA1* mRNA expression	Low vs. high (<or>optimal cut-off)	–	–	2.0 (1.1–3.7)	**0.028**
*BRCA2* mRNA expression	Low vs. high (<or>optimal cut-off)	1.9 (1.0–3.7)	0.061	1.9 (1.0–3.7)	0.058
B					
Age	Low vs. high (<or>median age)	–	–	2.1 (1.3–3.4)	**0.002**
FIGO stage	I/II vs. III/IV	2.3 (0.8–6.3)	0.119	1.1 (0.5–2.4)	0.812
Residual disease after surgery	No vs. yes	2.4 (1.3–4.7)	**0.008**	3.2 (1.7–6.0)	**<0.001**
*BRCA1* mRNA expression	Low vs. high (<or>optimal cut-off)	–	**–**	1.7 (0.8–3.5)	0.151
*BRCA2* mRNA expression	Low vs. high (or>optimal cut-off)	2.4 (1.0–5.7)	**0.045**	2.9 (1.2–6.8)	**0.015**

The significance level was determined by Cox regression analysis

*HR* hazard ratio. Bold values indicates $P \ \noexpand\lt$ 0.05

To answer the question whether the identified favorable survival in tumors with low *BRCA1*/*2-*mRNA expression may be interpreted by platinum-sensitivity we compared the expression levels in *BRCA1*-wildtype tumors from platinum-refractory and fully platinum-sensitive patients. We found statistically significant lower *BRCA1-* and *BRCA2* mRNA-expression levels in platinum-sensitive tumors (*P* = 0.004 and *P* = 0.045; Supplemental Fig. 1).

## Discussion

*BRCA1/2* belong to genes that play key roles in the homologous recombination repair, which represents the main mechanism to repair DNA double-strand breaks.^1^ While *BRCA1* is multifunctional, *BRCA2* functions almost exclusively in homologous recombination by recruiting an essential homologous recombination protein RAD51C to double-strand break sites.^19,20^ Our investigations revealed higher *BRCA1*/*2*-expression on the transcriptome level in OC tissues in comparison with non-neoplastic fallopian tube tissue. The cause of this finding may be the higher proliferation rate in malignant tissues which together with genetic instability may increase the need for more DNA damage repair. In accordance higher *BRCA1*/*2*-expression was also found in high-grade (Type II) tumors. This notion is supported by Gudas et al. who suggest that the upregulation of *BRCA1*-expression by steroid hormones is caused indirectly by increasing proliferation of breast cancer cells.^21^

Multivariate Cox-regression analysis showed a favorable OS for low *BRCA1*-expression in the whole cohort of included patients. For *BRCA2*-expression in the subgroup of high grade serous OC an independent, prognostic value in terms of PFS and OS was confirmed. These findings could be explained by a reduced capacity of DNA damage repair via homologous recombination in cancers with low *BRCA1/2* expression, enhancing the therapeutic effects of DNA-crosslinking agents such as platinum compounds. In fact, low *BRCA1/2* mRNA-expression in *BRCA1*-wildtype cancers was associated with fully platinum-sensitive disease and high expression was evidenced in platinum-refractory disease.

These *BRCA* mRNA-expression data are in line with the plethora of data showing that OC patients carrying a *BRCA1* or *2* germline mutation exhibit high responsiveness to platinum-based chemotherapy consecutively associated with an improved clinical outcome. In recurrent OC, similar beneficial therapeutical effects in *BRCA* mutation carriers have been reported for PARP-inhibitors and for trabectedin a drug that is crosslinking the DNA in the minor grove.

Until to date there are only very few studies on *BRCA*-expression in OC and these are only dealing with the expression of *BRCA1* but not with that of *BRCA2*. In a retrospective analysis of OC specimens obtained from patients included in the GOG-172 study comparing intraperitoneal (IP) with intravenous (i.v.) platinum/taxane chemotherapy, Lesnock et al. assessed BRCA1-expression on the protein level with regard to clinical outcome and responsiveness to chemotherapy considering especially the efficacy of the high loco-regional platinum doses reached by IP administration. The authors revealed that patients with cancers exhibiting BRCA1-immunostaining in less than 10% of the tumor cells was the only subgroup exhibiting a significant benefit in OS from a platinum-based IP chemotherapy.^22^ In addition, Carser et al. found also a strong response improvement to classical i.v. platinum-based chemotherapy in tumors with absent or low BRCA1-expression in immunohistochemistry. This effect was translated into a favorable PFS and OS in affected patients and the predictive value of BRCA1 immunostaining was confirmed in the multivariate analysis.^23^ These considerations were indirectly confirmed by Swisher et al., showing that in primary *BRCA1*-mutated OCs, recurrent platinum-resistant tumors exhibited secondary genetic changes within the *BRCA1* gene, which interestingly were accompanied by restored expression of BRCA1-protein.^24^ Furthermore, Quinn et al. reported from an in vitro and in vivo approach that inhibition of *BRCA1*-expression via siRNA knock-down leads to increased sensitivity to platinum therapy but impaired responsiveness to anti-microtubule agents such as taxanes. In a small series of patients, they corroborated their in vitro data by showing a significant improved OS in patients with tumors exhibiting low levels of *BRCA1-*mRNA.^25^ Also in this study *BRCA2*-expression has not been accessed.

In breast cancers exhibiting low *BRCA2* mRNA-levels, a significantly higher 5-year disease free survival rate was shown.^26^

Interestingly, our study emerged that in *BRCA1*-mutated OC the expression of *BRCA1*-transcripts was lower, but in contrary those of *BRCA2* were significantly higher as compared with *BRCA1*-wildtype cancers. Furthermore, also down regulation of *BRCA1*-transcripts by methylation of the *BRCA1*-promotor was associated with increased *BRCA2* mRNA levels. In contrast in cancers carrying a *BRCA2*-mutation, no up- or down-regulation of the *BRCA1*/*2* mRNA was found. However, the latter findings should be interpreted with caution due to the low number of *BRCA2*-mutated cancers within our cohort. The reason of the “compensatory” upregulation of *BRCA2*-mRNA in low *BRCA1*-expressing cancers remains speculative because there is no exact knowledge on how the BRCA protein expression is regulated either in normal or in malignant tissues. High *BRCA*-expression could determine a distinct phenotype with a high constitutive expression or could reflect a transitory upregulation triggered by various situations (e.g., proliferative or genomic stress). Thus, it is theoretically possible that the functional loss of multifunctional *BRCA1* is leading to genetically instable cancers requiring higher *BRCA2* recruitment for repeated double-strand break repair.

In *BRCA1* we found DNA-methylation in 11% of all tumors, which is in accordance with previously published data.^27^ We could not identify a prognostic relevance of *BRCA1* DNA-methylation for PFS and OS consistent with recent studies.^28,29^

Our data show that low *BRCA1*/*2* mRNA-expression confers platinum-hypersensitivity to OCs. As clinical studies in recurrent OC recently evidenced that the sensitivity of high grade serous OC to PARP-inhibitor maintenance therapy is particularly related to the response to the actual platinum-based chemotherapy,^30^ our data are tempting to speculate that *BRCA1*/*2* mRNA levels may be reliable biomarkers to also predict responsiveness of cancers to PARP-inhibitors. The same may be true for other drugs whose effectivity is related to platinum-sensitivity such as trabectedin.

## Electronic supplementary material

Supplemental Figure Legends

Supplemental Figure 1

## Data Availability

The datasets generated during and analyzed during the current study are available from the corresponding author on reasonable request.

## References

[1] Roy R, Chun J, Powell SN (2011). BRCA1 and BRCA2: different roles in a common pathway
of genome protection. Nat. Rev. Cancer.

[2] Saha S (2015). Decreased expression of BRCA2 accelerates sporadic
breast cancer progression. Indian J. Surg. Oncol..

[3] Siegel RL, Miller KD, Jemal A (2017). Cancer statistics, 2017. Cancer J. Clin..

[4] Holschneider CH, Berek JS (2000). Ovarian cancer: epidemiology, biology, and prognostic
factors. Semin. Surg. Oncol..

[5] Johannsson OT, Ranstam J, Borg A, Olsson H (1998). Survival of BRCA1 breast and ovarian cancer patients:
a population-based study from southern Sweden. J. Clin. Oncol..

[6] Risch HA (2001). Prevalence and penetrance of germline BRCA1 and BRCA2
mutations in a population series of 649 women with ovarian cancer. Am. J. Hum. Genet..

[7] Permuth-Wey J, Sellers TA (2009). Epidemiology of ovarian cancer. Methods Mol. Biol..

[8] Harter P (2016). BRCA1/2 mutations associated with progression-free
survival in ovarian cancer patients in the AGO-OVAR 16 study. Gyn. Oncol..

[9] Ledermann JA (2016). PARP inhibitors in ovarian cancer. Ann. Oncol..

[10] Pharoah PD, Easton DF, Stockton DL, Gayther S, Ponder BA (1999). Survival in familial, BRCA1-associated, and
BRCA2-associated epithelial ovarian cancer. United Kingdom Coordinating
Committee for Cancer Research (UKCCCR) Familial Ovarian Cancer Study
Group. Cancer Res..

[11] Sabatier R (2016). Ovarian cancer patients at high risk of BRCA mutation:
the constitutional genetic characterization does not change
prognosis. Fam. Cancer.

[12] Kotsopoulos J (2016). Ten-year survival after epithelial ovarian cancer is
not associated with BRCA mutation status. Gyn. Oncol..

[13] Goebel G (2012). Elevated mRNA expression of CHAC1 splicing variants is
associated with poor outcome for breast and ovarian cancer
patients. Br. J. Cancer.

[14] Notaro S (2016). Evaluation of folate receptor 1 (FOLR1) mRNA
expression, its specific promoter methylation and global DNA hypomethylation in
type I and type II ovarian cancers. BMC Cancer.

[15] Weisenberger DJ (2006). CpG island methylator phenotype underlies sporadic
microsatellite instability and is tightly associated with BRAF mutation in
colorectal cancer. Nat. Genet..

[16] Press JZ (2008). Ovarian carcinomas with genetic and epigenetic BRCA1
loss have distinct molecular abnormalities. BMC Cancer.

[17] Eads CA (2001). Epigenetic patterns in the progression of esophageal
adenocarcinoma. Cancer Res..

[18] Youden WJ (1950). Index for rating diagnostic tests. Cancer.

[19] Moynahan ME, Pierce AJ, Jasin M (2001). BRCA2 is required for homology-directed repair of
chromosomal breaks. Mol. Cell.

[20] Takaoka M, Miki Y (2017). BRCA1 gene: function and deficiency. Int. J. Clin. Oncol..

[21] Gudas JM, Nguyen H, Li T, Cowan KH (1995). Hormone-dependent regulation of BRCA1 in human breast
cancer cells. Cancer Res..

[22] Lesnock JL (2013). BRCA1 expression and improved survival in ovarian
cancer patients treated with intraperitoneal cisplatin and paclitaxel: a
Gynecologic Oncology Group Study. Br. J. Cancer.

[23] Carser JE (2011). BRCA1 is both a prognostic and predictive biomarker of
response to chemotherapy in sporadic epithelial ovarian cancer. Gyn. Oncol..

[24] Swisher EM (2008). Secondary BRCA1 mutations in BRCA1-mutated ovarian
carcinomas with platinum resistance. Cancer Res..

[25] Quinn JE (2007). BRCA1 mRNA expression levels predict for overall
survival in ovarian cancer after chemotherapy. Clin. Cancer Res..

[26] Egawa C, Miyoshi Y, Taguchi T, Tamaki Y, Noguchi S (2002). High BRCA2 mRNA expression predicts poor prognosis in
breast cancer patients. Int. J. Cancer.

[27] Cunningham JM (2014). Clinical characteristics of ovarian cancer classified
by BRCA1, BRCA2, and RAD51C status. Sci. Rep..

[28] Yang D (2011). Association of BRCA1 and BRCA2 mutations with
survival, chemotherapy sensitivity, and gene mutator phenotype in patients with
ovarian cancer. JAMA.

[29] Ruscito I (2014). BRCA1 gene promoter methylation status in high-grade
serous ovarian cancer patients—a study of the tumour Bank ovarian cancer (TOC)
and ovarian cancer diagnosis consortium (OVCAD). Eur. J. Cancer.

[30] Mirza MR (2016). Niraparib maintenance therapy in platinum-sensitive,
recurrent ovarian cancer. N. Engl. J. Med..

