# Supplementary material for: BRCA1 and BRCA2 mRNA-expression prove to be of clinical impact in ovarian cancer
Source: Br J Cancer. 2018 Aug 15;119(6):683–92. doi: 10.1038/s41416-018-0217-4 (PMC6173779; doi:10.1038/s41416-018-0217-4)
Supplement: Supplementary file 1 — Supplemental Figure Legends [file 41416_2018_217_MOESM1_ESM.docx]

**Supplemental Figure Legends**

**Supplemental Fig. 1: *BRCA1/2* mRNA-expression and platinum-sensitivity in *BRCA1*-wildtype tumors.** (**a**) *BRCA1* and (**b**) *BRCA2* mRNA-expression in fully platinum-refractory and platinum-sensitive tumors.
