# Supplementary figures and images for: BRCA1 and BRCA2 mRNA-expression prove to be of clinical impact in ovarian cancer
Source: Br J Cancer. 2018 Aug 15;119(6):683–92. doi: 10.1038/s41416-018-0217-4 (PMC6173779; doi:10.1038/s41416-018-0217-4)

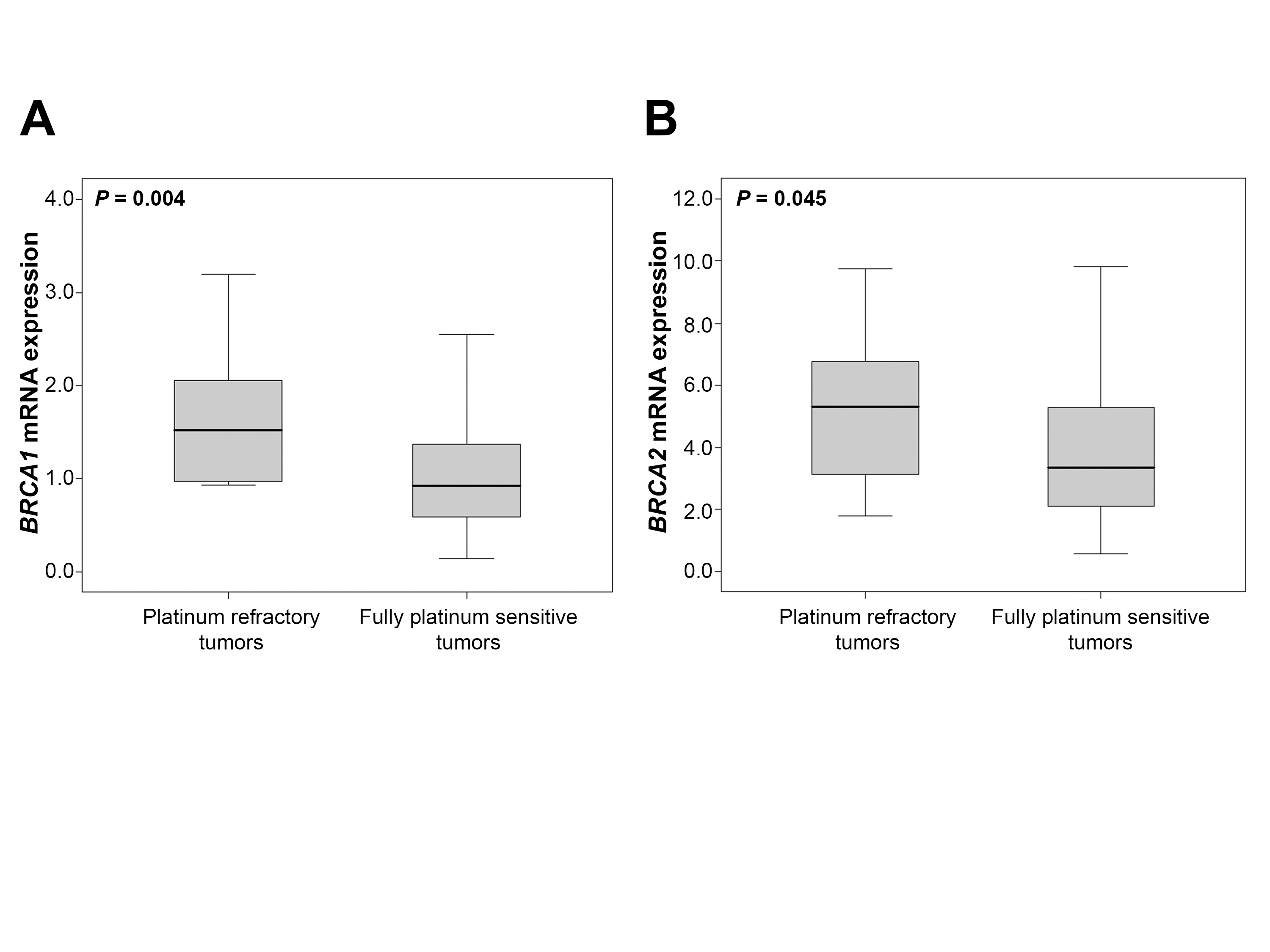

Supplement: Supplementary file 2 — Supplemental Figure 1 [file 41416_2018_217_MOESM2_ESM.tif]
